# Dermatoses in Postmenopausal Women in a Tertiary Health Care Center of Bihar: A Prospective Cross-Sectional Study

**DOI:** 10.7759/cureus.41587

**Published:** 2023-07-09

**Authors:** U. K. Pallavi, Rajesh Sinha, Kranti Chandan Jaykar, Subhasree Sarkar, Tajwar Yasmeen, Dipali Prasad

**Affiliations:** 1 Dermatology, Indira Gandhi Institute of Medical Sciences, Patna, IND; 2 Dermatoiogy, Indira Gandhi Institute of Medical Sciences, Patna, IND; 3 Community Medicine, Indira Gandhi Institute of Medical Sciences, Patna, IND; 4 Obstetrics and Gynacology, Indira Gandhi Institue of Medical Sciences, Patna, IND

**Keywords:** genital dermatosis, lichen sclerosus et atrophicus, oral lichen planus, dermatophytosis, estrogen deficiency, menopause, post-menopausal dermatosis

## Abstract

Background

Postmenopausal women comprise a very unique population with various dermatological, genital, and oral dermatoses due to the various physiological, age-related, and hormonal changes in this period, which have not yet been studied extensively, especially in India.

Aims and objectives

The aim of the study was to study the various epidemiological and clinical patterns of postmenopausal dermatosis.

Material and methods

We conducted a single-center observational cross-sectional study on 223 postmenopausal women attending the dermatology outpatient department (OPD) with various dermatological concerns to understand the various clinical patterns and presentations of postmenopausal dermatoses. Women were interviewed face to face using a pre-designed, pre-tested questionnaire. A detailed history followed by general physical, systemic, and cutaneous examination was done, along with investigations wherever needed.

Results

A total of 223 postmenopausal women were enrolled in our study, with a mean age group of 58.4 ± 5.1 years. The mean age of menopause in our study was 48.7 ± 3.8 years. In our study, 186 (83.4%) women had cutaneous dermatosis, 65 (29.1%) had genital dermatosis, 23 (10.3%) had oral mucosa involvement, 75 (33.6%) had hair disorders, and 58 (26%) had nail disorders.

Limitation

The limitation of our study is that it is a single-center study, and women with active HIV or hepatitis infection or known malignancy were excluded from the study.

Conclusion

A broader understanding of the diverse dermatological concerns of postmenopausal women would enable dermatologists to be better equipped to identify and treat postmenopausal dermatosis as well as provide better support to women going through this phase of life.

## Introduction

Postmenopausal women (physiological menopause, early physiological menopause, or surgical menopause) comprise a very unique population with various dermatological, genital, and oral dermatoses due to the various physiological, age-related, and hormonal changes in this period, which have not yet been studied extensively especially in India [1]. Menopause is defined as the permanent, irreversible cessation of menses (not having a menstrual period for 12 consecutive months) brought about by a decline in ovarian follicular activity, usually caused due to a decline in estrogen levels [2]. There is a multitude of factors at play in menopause leading to dermatosis, which impacts physical, psychological, and sexual well-being in postmenopausal women [3].

## Materials and methods

Study type and period

This is a cross-sectional study that was carried on from October 2021 to September 2022 after ethical approval from the Institutional Human Research Ethics Committee of Indira Gandhi Institute of Medical Sciences.

Study design

All postmenopausal women with dermatological concerns attending the dermatology outpatient department OPD, as well as cases referred by gynecology OPD, were interviewed face-to-face using a pre-designed, pre-tested questionnaire after taking proper written consent in vernacular language. Data related to socio-demographic variables and age of menarche, as well as menopause, was recorded. A detailed history followed by a general physical and systemic examination was done. Relevant past history of any systemic diseases as well as drug intake and allergic history was taken. A thorough cutaneous examination of the skin, hair, and nails was done for physiological as well as pathological changes. A genital examination was done in patients with related complaints. Investigations like KOH smear, Wood's lamp examination, bacterial and fungal culture, slit-skin smear, skin biopsy, Tzanck smear, and blood investigations were done wherever needed. Diagnosis was made by a dermatologist. Relevant scores using established scoring systems pertaining to specific diseases were also done where needed. Women with active HIV, hepatitis, tuberculosis infection, or known malignancy were excluded from the study.

Statistical analysis

The data was recorded and analysis was done using SPSS software (IBM Inc., Armonk, New York) and Microsoft Excel (Microsoft, Redmond, Washington); relevant mean, standard deviation, and percentages were calculated.

## Results

**Figure 1:**
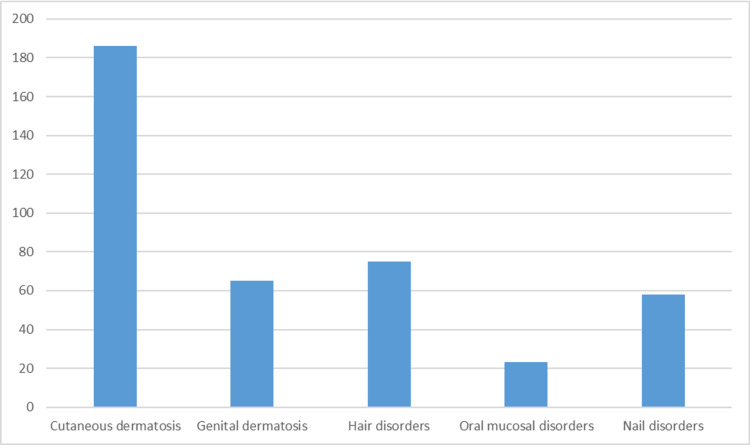
Frequency of different types of dermatosis

A total of 223 postmenopausal women between the ages of 44 years to 86 years were enrolled in our study, with a mean age of 58.4± 5.1 years. There were 88 (39.5%) women in the age group of 51-60 years and 73 (32.7%) women in the age group of 61-70 years, followed by 34 (15.2%) women in the age group of 71-80 years, and 23 (10.3%) women in the age group of 40-50 years, and only five (2.2%) women in the age group of greater than 80 years. The mean age of menopause in our study was 48.7 ± 3.8 years. The majority of the women were of the lower middle (37.7 %) and upper lower (28.3%) socio-economic status. Out of the total 223 women enrolled, 51 (22.8%) women had co-morbidities, which included type 2 diabetes mellitus, hypertension, coronary artery disease, and hypothyroidism in 24 (10.8%), 31 (13.9%), 6 (2.7%) and 17 (7.6%), respectively, that were not exclusive of each other. Diabetes was controlled in 17 patients and uncontrolled in seven patients. In our study, 186 (83.4%) women had cutaneous dermatosis, 65 (29.1%) had genital dermatosis, 23 (10.3%) had oral mucosa involvement, 75 (33.6%) had hair disorders, and 58 (26%) had nail disorders (Figure 1). The most common cutaneous complaint and diagnoses were generalized pruritus (60.8%) and superficial dermatophytosis in 39 (21.0%) women. The most common genital dermatosis was tinea cruris in 19 (29.2%), candidal intertrigo in nine (13.8%), lichen simplex chronicus in nine (13.8%), and lichen sclerosus et atrophicus in seven (10.8%) patients. The most common oral mucosal complaint was a burning sensation of the mouth (78%), and the most common diagnosis was oral lichen planus in nine (39.1%) patients. The most common hair disorder was female pattern hair loss in 35 (46.7%) women, and the most common nail disorder was onychomycosis in 25 (43.1%) women.

## Discussion

Menopause occurs due to the cessation of normal follicular maturation and ovulation. Studies have found that in most women, menopause occurs between the ages of 50-55 years, with an average age of 51.5 years; however, the estimated mean age of menopause is 46 years in India which is lower than in Caucasians [2]. This has been attributed to several factors like marital status, increased parity, lifestyle, dietary habits, heredity, and a comparative increase in the body mass index in Indian women [3]. There is also a subset of women who undergo premature menopause before the age of 40 and some women who continue to menstruate till 60 years of age. The age at which a woman achieves menopause depends on a number of factors like heredity, nutrition, socioeconomic factors, lifestyle, exposure to toxins, and smoking [4].

Menopausal women undergo physiological, as well as age-related atrophy of skin and appendages, genital system, and breasts. External factors like sun exposure, lifestyle, nutrition, smoking, and other toxins, as well as intrinsic telomere shortening, lead to skin aging due to increased oxidative stress [5-6]. There is also a decrease in collagen and fibroblast content, low collagen synthesis, and slow turnover, along with a decrease in skin thickness, suppleness, resilience, and pliability with age [7]. Sebaceous and sweat glands also undergo slow atrophy leading to xerosis cutis. Estrogen receptors are widely distributed in the epidermis and dermis, and a fall in estrogen levels in menopause leads to significant skin changes [8]. The glandular tissue of breasts and uterus get replaced with an increased amount of fibrous tissue, and the vulvovaginal as well as urinary tract epithelium atrophy leading to increased dryness, itching, burning, chances of infection and dyspareunia [9]. Greying of hair and conversion of terminal to vellus hair occurs in the scalp, and the density of the scalp as well as pubic hair decreases. Salivary flow rates are dependent on estrogen status, which might lead to discomfort, dryness, and a burning sensation in menopause [10]. There are also several skin disorders that are not specific to menopause but are found to be increased in menopause, as well as some dermatoses which occur due to hormonal replacement therapy in menopause, like hirsutism, acne, and androgenetic alopecia. With the increase in female life expectancy in India, which according to the World Health Organization 2011 health statistics, is up to 73 years by 2021, the number of women going through menopause each year is rising, and therefore menopause and its associated dermatological symptoms are increasingly becoming a key area of interest [11].

Menopause is associated with hormonal changes, the most important being the reduction in estrogen levels and the increase in follicle-stimulating hormone (FSH) levels [12]. These hormonal changes, particularly the decrease in the level of estrogen, play an important role in the alterations of the normal physiology and composition of skin and mucosa in menopause. There are two types of estrogen receptors- alpha receptors that are expressed on fibroblasts and macrophages, and beta receptors that are present on basal keratinocytes, melanocytes, and dendritic cells [13]. It has been reported that a fall in estrogen levels leads to a decrease in epidermal differentiation and slower maturation, as well as a decrease in collagen, fatty acids, and matrix synthesis.

It is an important phase of a woman's life, and it is imperative to understand the diverse dermatological concerns better. Our study was conducted in a tertiary care center in Bihar which is a part of Eastern India and has higher temperatures and humidity, and our study comprised majorly of women from lower middle and upper lower socioeconomic status. The strength of this study is that it was conducted in a tertiary care center that is centrally located and accessible to different strata of the population.

**Figure 2:**
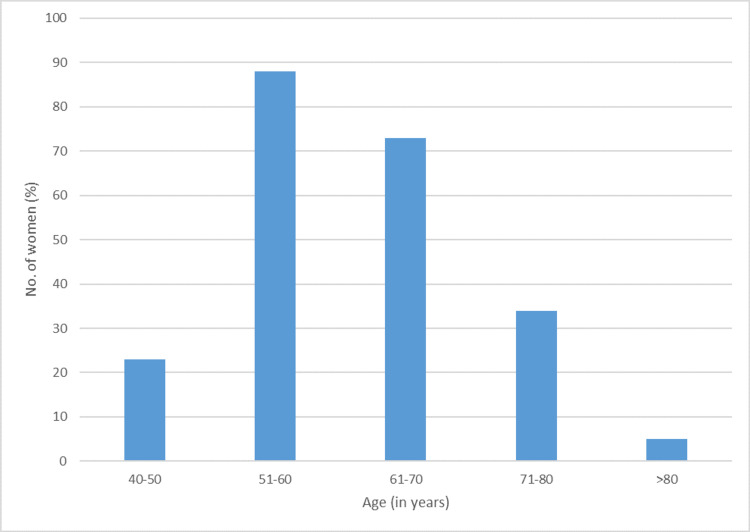
Age distribution

In our study, we enrolled a total of 223 women, with ages ranging from 44 years to 86 years, with 88 (39.5%) women in the age group of 51-60 years, 73 (32.7%) women in the age group of 61-70 years, 34 (15.2%) women in the age group of 71-80 years, 23 (10.3%) women in the age group of 40-50 years, and only five (2.2%) women in the age group of greater than 80 years. The mean age of women in our study was 58.4± 5.1 years (Figure 2).

The majority of the women were of lower middle and upper lower socioeconomic status, 84 (37.7 %) and 63 (28.3%), respectively. Most of the women were housewives (79.8%), and only a few were office workers (16.6%) and laborers (3.6%). Out of 223, 212 women were currently married, nine were currently widowed, and only two were unmarried (Table 1).

**Table 1:** Socio-demographic data

	No. of women	Percentage (%)
Age group (years)		
40-50	23	10.3
51-60	88	39.5
61-70	73	32.7
71-80	34	15.2
>80	5	2.2
Age at menopause (years)		
30-40	2	0.9
41-50	118	52.9
51-60	102	45.7
61-70	1	0.4
Marital status		
Married	212	95.1
Widowed (currently)	9	4
Unmarried	2	0.9
Separated	0	0
Occupation		
Housewife	178	79.8
Office worker	37	16.6
Labourer	8	3.6
Socioeconomic status		
Upper	5	2.2
Upper-middle	14	6.3
Lower-middle	84	37.7
Upper-lower	63	28.3
Lower	57	25.6
Co-morbidities		
Diabetes mellitus (Type 2)	24	10.8
Hypertension	31	13.9
Coronary artery disease	6	2.7
Hypothyroidism	17	7.6

Maximum women reported to have achieved menopause between the age of 41-50 years and 51-60 years, 118 (52.9%) and 102 (45.7%), respectively. The mean age of menopause in our study was 48.7 ± 3.8 years. In previous studies by Aboobacker et al. [12] and Dasgupta et al. [14], the mean age of menopause was 46.24 (±3.38) years and 46.14 (±4.47) years, respectively. Similar studies by Sharma et al. [2] and Kaulageka et al. [15] in Jammu and Maharashtra found the mean age of menopause as 47.53 years and 45.8 (±4.3) years, respectively. In our study, none of the women were smokers; however, 23 (10.3%) women admitted to tobacco and betel leaf ('paan') chewing. The limitation of our study is that it is a single-center study, and women with active HIV, hepatitis infection, or known malignancy were excluded from the study.

Out of the total 223 women enrolled, 51 (22.8%) women had co-morbidities, which included type 2 diabetes mellitus, hypertension, coronary artery disease, and hypothyroidism in 24 (10.8%), 31 (13.9%), 6 (2.7%) and 17 (7.6%), respectively, but not exclusive of each other. Diabetes was controlled in 17 patients and uncontrolled in seven patients. These co-morbidities were found to be more associated with a few dermatoses in particular, which have been discussed below. Studies have shown that a decrease in estrogen levels has been associated with an increased risk of cardiac events. A relationship between menopause and hypertension, as well as serum cholesterol, has been suggested by Weiss [16].

**Figure 3:**
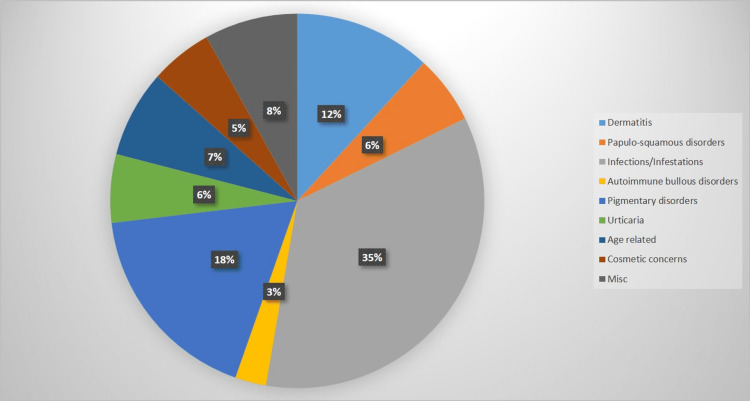
Cutaneous dermatoses (groups)

A total of 186 (83.4%) out of 223 women enrolled complained of having cutaneous dermatosis. The most common diagnosis was superficial dermatophytosis (21.0%), and the most common complaint was generalized pruritus (60.8%) (Table 2). This is similar to the study by Pariath et al., where the most common non-genital dermatosis out of 102 post-menopausal women was superficial dermatophytosis (16.33%), lichen planus (11.22%), psoriasis (9.18%), and eczema (8.16%) [3]. However, this was in contrast to a study conducted by Aboobacker et al., where the most common dermatoses were eczematous dermatitis (23.9%) followed by urticaria (12.3%) and papulosquamous disorders (10.9%) [12]. Tinea corporis was the most common type of dermatophytosis in our study. A higher temperature and humidity, which leads to increased sweating, as well as the type of clothing, socioeconomic status, overcrowding, hygiene, and lifestyle, might contribute to superficial dermatophytosis. A few cases of resistant dermatophytosis were closely related to uncontrolled diabetes mellitus. Cases of herpes zoster and postherpetic neuralgia, intertrigo, pityriasis versicolor, Hansen's disease (two borderline tuberculoid and one neuritic), and scabies were diagnosed in nine (4.8%), six (3.2%), four (2.2%), three (1.6%), and two (1.1%) patients, respectively. Intertrigo was seen to be closely related to diabetes mellitus. Dermatitis was seen in 22 (11.8%) patients, the most common being asteatotic eczema (3.2%) and allergic contact dermatitis (2.7%). Among papulosquamous disorders, psoriasis (3.2%) was present in six patients, and lichen planus was present in five (2.7%) patients. The most common type of psoriasis was chronic plaque psoriasis with an average Psoriasis Area Severity Index (PASI) score of eight. Autoimmune bullous disorders were seen in five patients, three (1.6%) of pemphigus vulgaris and two (1.1%) of bullous pemphigoid. Urticaria was diagnosed in 11 (5.9%) patients, and out of the total 11, hypothyroidism was present in three patients and two patients had diabetes mellitus and hypothyroidism both. Among hyperpigmentary disorders, melasma was the most common and diagnosed in eight (4.3%) patients. The most common type was epidermal and in malar distribution, and usually, the patients had an associated history of long duration of sun exposure. Vitiligo was diagnosed in five (2.7%) patients, and the most common form was vitiligo vulgaris. Idiopathic guttate hypomelanosis was also diagnosed in five patients. Age-related disorders seen in our study were senile pruritus, senile comedones, wrinkles, and infra-orbital laxity. Senile pruritus was present in 10 (5.8%) patients, and senile comedones around the peri-orbital area were present in four (2.3%) patients (Table 3, Figure 3). Wrinkles and infra-orbital laxity were concerns that seven (3.8%) and three (1.6%) women presented with; however, it was identified in 44.8% and 21% of women, respectively. Environmental exposure to sunlight, pollution, lifestyle, and repetitive muscle movements, along with a decrease in collagen and fibroblast content and loss of skin pliability and suppleness with aging, seem to contribute to these changes.

**Table 2:** Cutaneous dermatoses (individual)

Cutaneous dermatosis	No. of women	Percentage (%)
Superficial dermatophytosis	39	21.0
Asteatotic eczema	6	3.2
Allergic contact dermatitis	5	2.7
Irritant contact dermatitis	3	1.6
Psoriasis	6	3.2
Lichen planus	5	2.7
Melasma	8	4.3
Melanocytic nevus	3	1.6
Seborrheic keratosis	2	1.1
Acrochordon	4	2.2
Opportunistic infections	2	1.1
Hansen's disease	3	1.6
Vitiligo	5	2.7
Diabetic dermatopathy	2	1.1
Pemphigus vulgaris	3	1.6
Bullous pemphigoid	2	1.1
Herpes zoster and postherpetic neuralgia	9	4.8
Acanthosis nigricans	2	1.1
Hirsutism	2	1.1
Lichen/ macular amyloidosis	5	2.7
Intertrigo	6	3.2
Senile comedones	4	2.2
Senile pruritus	10	5.4
Keloid/ hypertrophic scar	3	1.6
Photodermatitis	2	1.1
Chronic urticaria	8	4.3
Acute urticaria	3	1.6
Keratoderma climactericum	4	2.2
Scabies	2	1.1
Stasis dermatitis	2	1.1
Ulcer	2	1.1
Pityriasis versicolor	4	2.2
Idiopathic guttate hypomelanosis	5	2.7
Peri-orbital melanosis	5	2.7
Infra-orbital laxity	3	1.6
Wrinkles	7	3.8
Total	186	100.0

**Table 3:** Cutaneous dermatoses (groups)

Groups	No. of women	Percentage(%)
Dermatitis	22	11.8
Papulosquamous disorders	11	5.9
Infections/ infestations	65	34.9
Autoimmune bullous disorders	5	2.7
Pigmentary disorders	28	17.7
Urticaria	11	5.9
Age-related	14	7.5
Cosmetic concerns	10	5.4
Miscellaneous	15	8.1
Total	171	100.0

In our study, 65 (29.1%) patients had genital involvement, and the most common diagnosis was tinea cruris in 19 (29.2%) and candidal intertrigo in nine (13.8%) patients. This was significantly higher than the study by Pariath et al., where the frequency was 15.55%, and a study by Kaur and Kalsy, where it was 7.5% [3,17]. The most common genital complaints encountered in our study were pruritus and genital lesions. Diabetes was closely related to candidal intertrigo and resistant tinea cruris infections. Lichen simplex chronicus was diagnosed in nine (13.8%) patients, and lichen sclerosus et atrophicus was diagnosed in seven (10.8%) patients. In a study by Pariath et al., the prevalence of genital lichen simplex chronicus was found to be 15.5%, and in a study by Kaur and Kalsy, it was 42.5% [3,17]. A few cases of genital lichen planus, vitiligo, herpes genitalis, and warts were encountered in our study too (Table 4).

**Table 4:** Genital dermatoses

Genital dermatosis	No. of women	Percentage (%)
Lichen sclerosus et atrophicus	7	10.8
Lichen simplex chronicus	9	13.8
Genital lichen planus	3	4.6
Atrophic vaginitis	3	4.6
Tinea cruris	19	29.2
Opportunistic infections	2	3.1
CandidalIntertrigo	9	13.8
Vitiligo	2	3.1
Candidiasis	6	9.2
Herpes genitalis	2	3.1
Warts	2	3.1
Ulcer	1	1.5
Total	65	100.0

In our study, 23 (10.3%) patients had oral complaints, the most common complaint being a burning sensation of the mouth (78%), and the most common diagnosis was oral lichen planus of the reticulate variety in nine (39.1%) patients (Table 5). This was higher than the studies by Pariath et al. and Mohan et al., where the frequency of oral lichen planus was 13.75% and 10.91%, respectively [3,18]. This is significantly higher than the prevalence in the general population, and a fall in the levels of estrogen after menopause and its effect on cell-mediated immunity has been implicated in the pathogenesis. Estrogen is also reported to impact salivary flow rates, and therefore its lower levels might lead to oral discomfort, dryness, and a burning sensation.

**Table 5:** Oral cavity dermatoses

Oral cavity dermatosis	No. of women	Percentage (%)
Lichen planus	9	4.0
Pemphigus vulgaris	2	0.9
Aphthous ulcers	4	1.8
Dryness of mouth	5	2.2
Burning mouth syndrome	3	1.3

In our study, 75 (33.6%) women had complaints of hair loss, and non-scarring alopecia was present in 96% of the patients. The most common diagnosis was female pattern hair loss and diffuse hair loss in 46.7% and 32% of patients, respectively (Table 6). Estrogen, which influences hair growth and hair cycle, might be considered contributory to the increased incidence of female pattern hair loss in menopause. Lichen plano-pilaris causing scarring alopecia was seen in only three (4%) women.

**Table 6:** Hair disorders

Hair disorders	No. of women	Percentage (%)
Diffuse hair loss	24	32.0
Male pattern hair loss	11	14.7
Female pattern hair loss	35	46.7
Lichen planopilaris	3	4.0
Alopecia areata	2	2.7
Total	75	100.0

In our study, 58(26%) women had nail abnormalities, and the most common complaint and diagnosis was yellowish discoloration of nails and onychomycosis (43.1%), respectively (Table 7). The most common type was distal subungual onychomycosis, and toenails were more commonly affected. Both onychomycosis and paronychia were associated with chronic wet work and diabetes mellitus, both controlled and uncontrolled blood sugar levels. In the study by Pariath et al., the percentage of onychomycosis was 14.67% which is significantly lower than our study [3]. Onychorrhexis, onychoschizia, and nail pitting were also seen in a few women.

**Table 7:** Nail disorders

Nail disorders	No. of women	Percentage (%)
Onychomycosis	25	43.1
Paronychia	8	13.8
Nail pitting	5	8.6
Subungual hyperkeratosis	3	5.2
Onychoschizia	6	10.3
Onychorrhexis	8	13.8
Pterygium	3	5.2
Total	58	100.0

## Conclusions

There are a plethora of skin, mucosal, hair, and nail changes seen in menopause due to physiological changes, aging, hormonal changes, or hormonal therapy, which might significantly affect the quality of life in postmenopausal women. A broader understanding of the diverse dermatological concerns of postmenopausal women would enable dermatologists to be better equipped to identify and treating such dermatoses as well as provide better support to women going through this phase of life.
